# Supplementary material for: Targeting the mevalonate or Wnt pathways to overcome CAR T-cell resistance in TP53-mutant AML cells
Source: EMBO Mol Med. 2024 Feb 14;16(3):3. doi: 10.1038/s44321-024-00024-2 (PMC10940689; doi:10.1038/s44321-024-00024-2)
Supplement: Supplementary file 1 — Appendix [file 44321_2024_24_MOESM1_ESM.pdf]

Appendix to

Targeting the mevalonate or Wnt pathways to overcome CAR T-cell resistance in *TP53*-mutant AML cells

Jan Mueller[1*], Roman R. Schimmer[1*], Christian Koch[1], Florin Schneiter[2], Jonas Fulin[1], Veronika Lysenko[1], Christian Pellegrino[1], Nancy Klemm[1], Norman Russkamp[1], Renier Myburgh[1], Laura Volta[1], Alexandre P.A. Theocharides[1], Kari J. Kurppa[3], Benjamin L. Ebert[4], Timm Schroeder[2], Markus G. Manz[1#] and Steffen Boettcher[1#]

[1]Department of Medical Oncology and Hematology, University of Zurich and University Hospital Zurich, Zurich, Switzerland

[2]Department of Biosystems Science and Engineering, ETH Zurich, Basel, Switzerland

[3]Institute of Biomedicine and Medicity Research Laboratories, University of Turku, Turku, Finland

[4]Department of Medical Oncology, Dana-Farber Cancer Institute, Boston, MA

Running Title: CAR T-cell resistance in *TP53*-mutant AML

Corresponding Author:
Steffen Boettcher, MD
Department of Medical Oncology and Hematology
University of Zurich and University Hospital Zurich
Raemistrasse 100
CH-8091 Zurich
Switzerland
Phone: +41-43-253 9299
steffen.boettcher@usz.ch

*J.M. and R.R.S. contributed equally to this study. #M.G.M. and S.B. jointly directed this study.

APPENDIX FIGURES AND TABLES

Appendix Figure S1	page 3
Appendix Figure S2	page 4
Appendix Figure S3	page 5
Appendix Figure S4	page 6
Appendix Figure S5	page 7
Appendix Figure S6	page 8
Appendix Table S1	page 9
Appendix Table S2	page 9
Appendix Table S3	page 9
Appendix Figure legends	pages 10-11

Appendix Figure S1

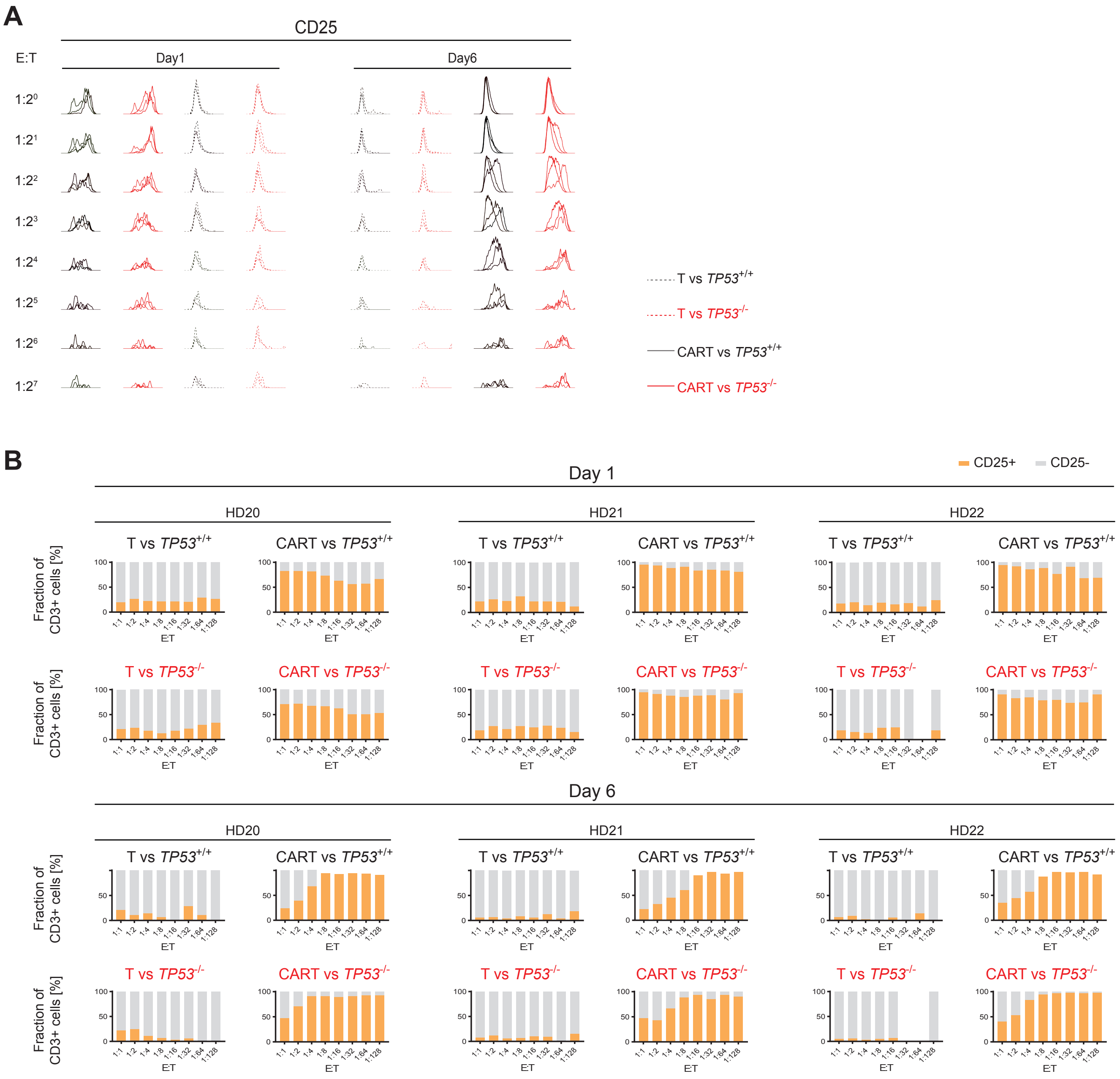

Appendix Figure S2

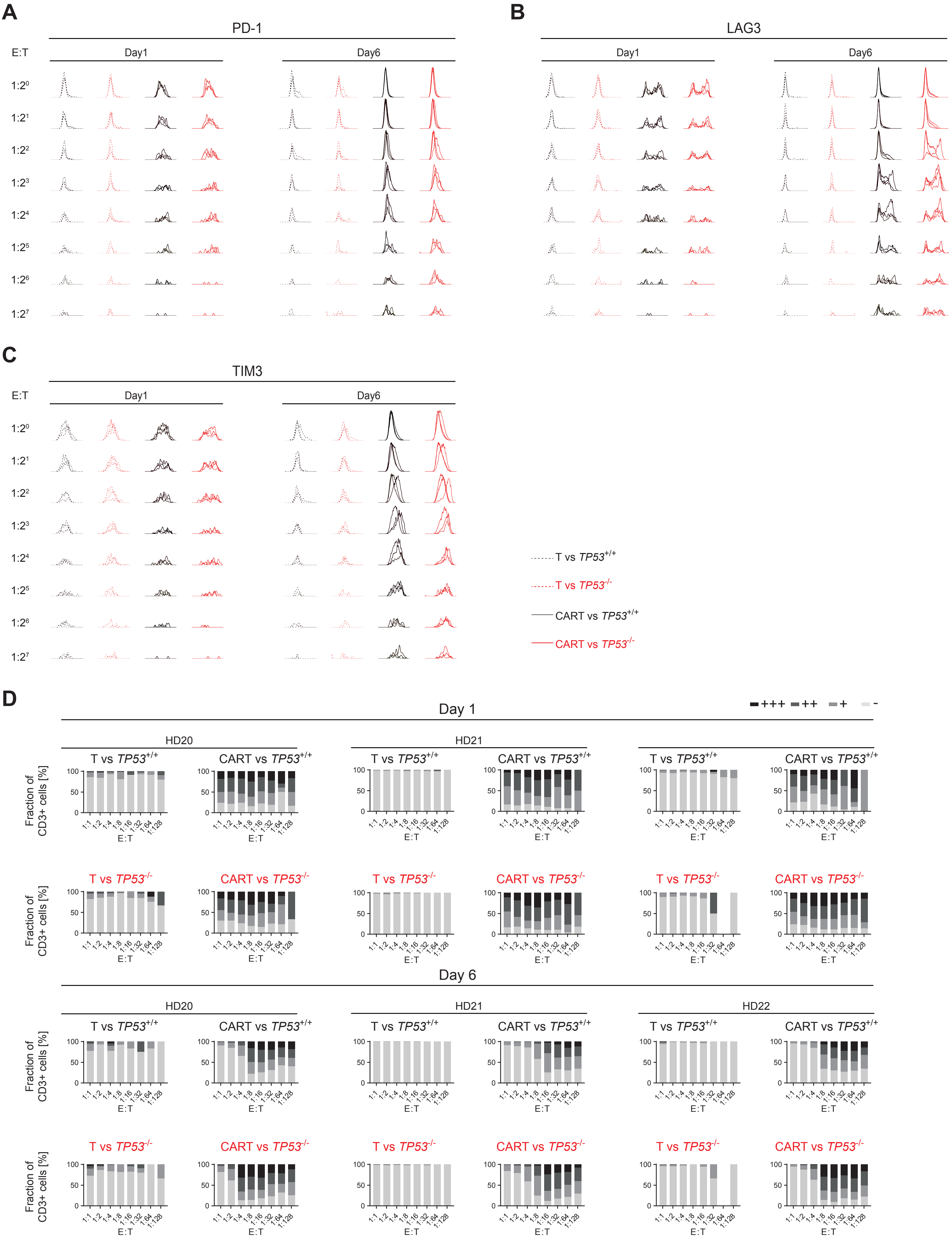

Appendix Figure S3

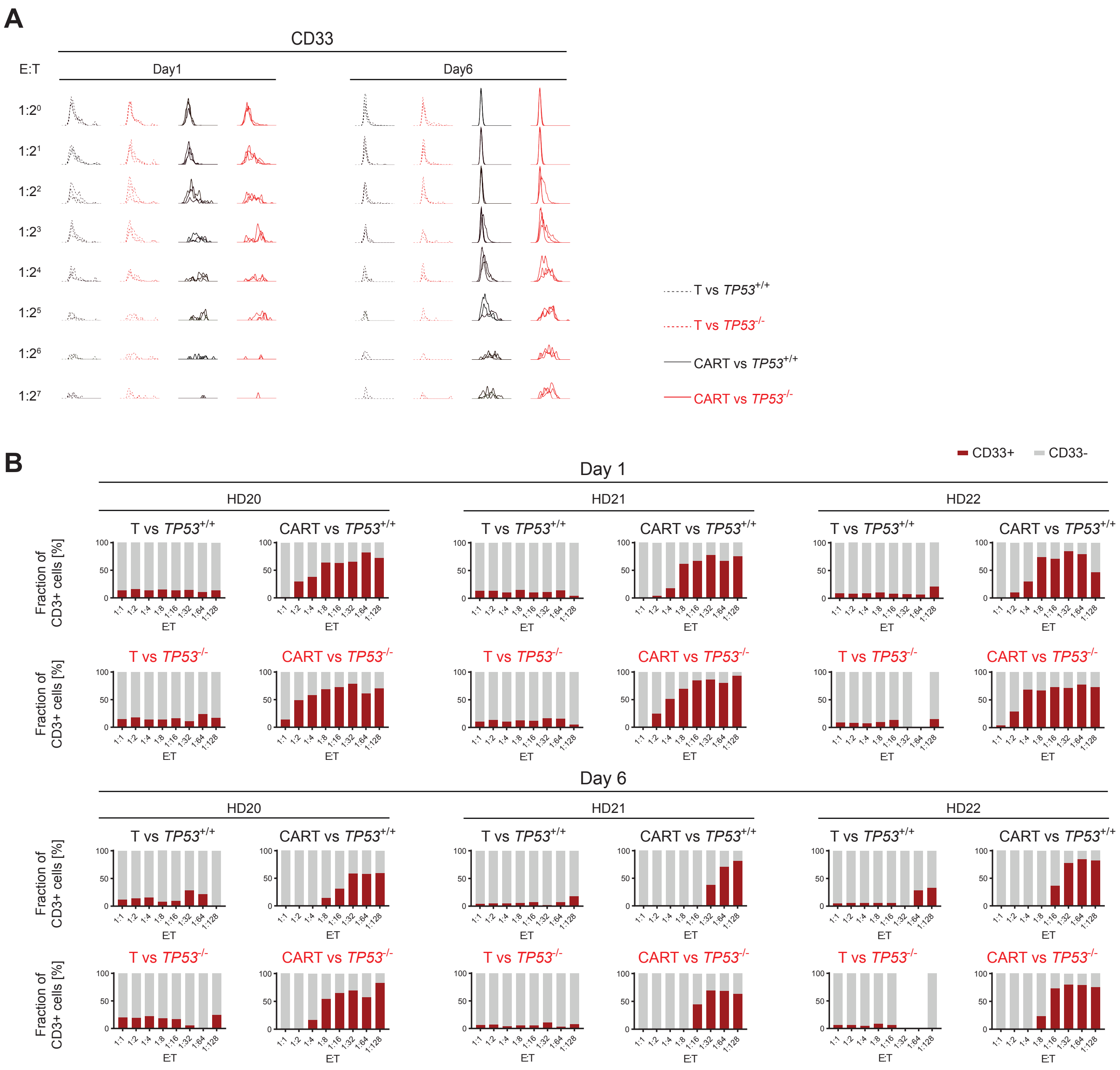

Appendix Figure S4

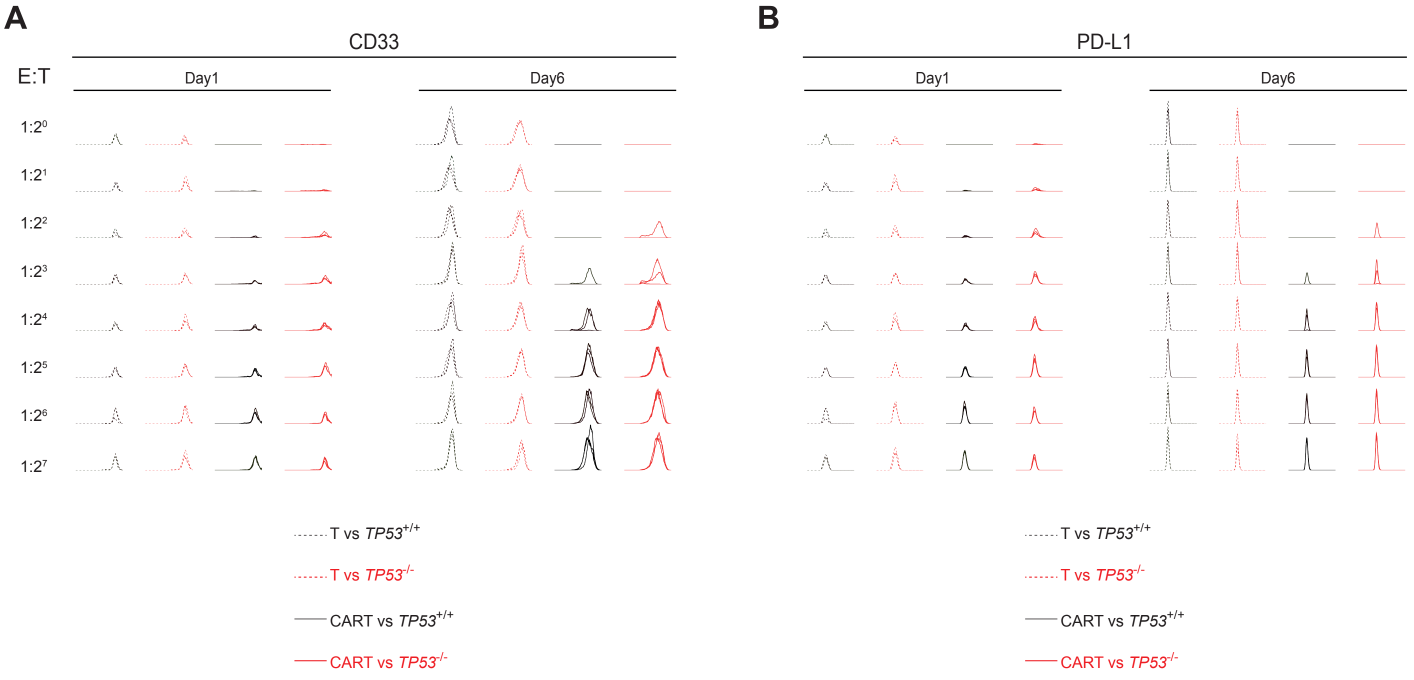

Appendix Figure S5

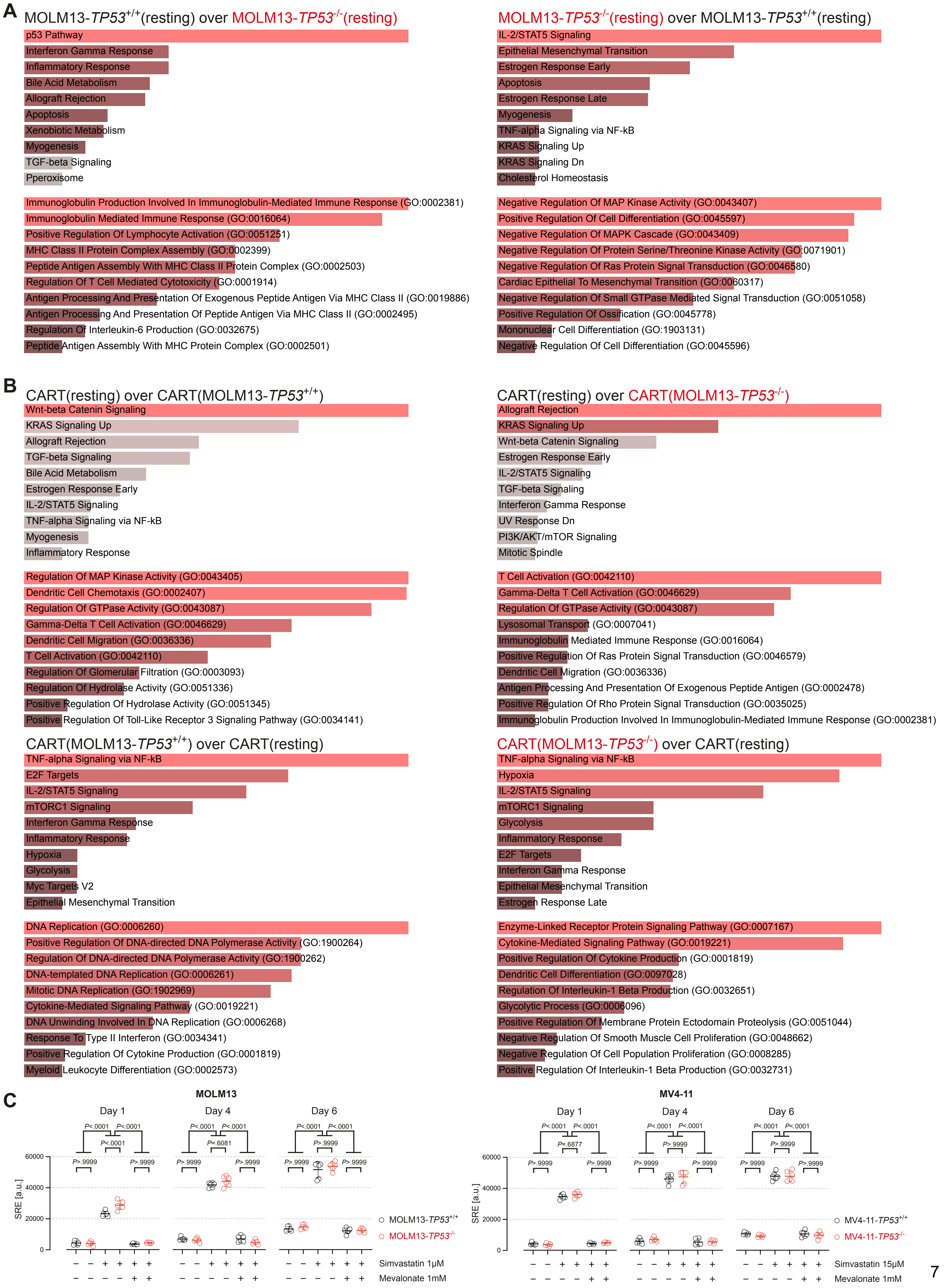

Appendix Figure S6

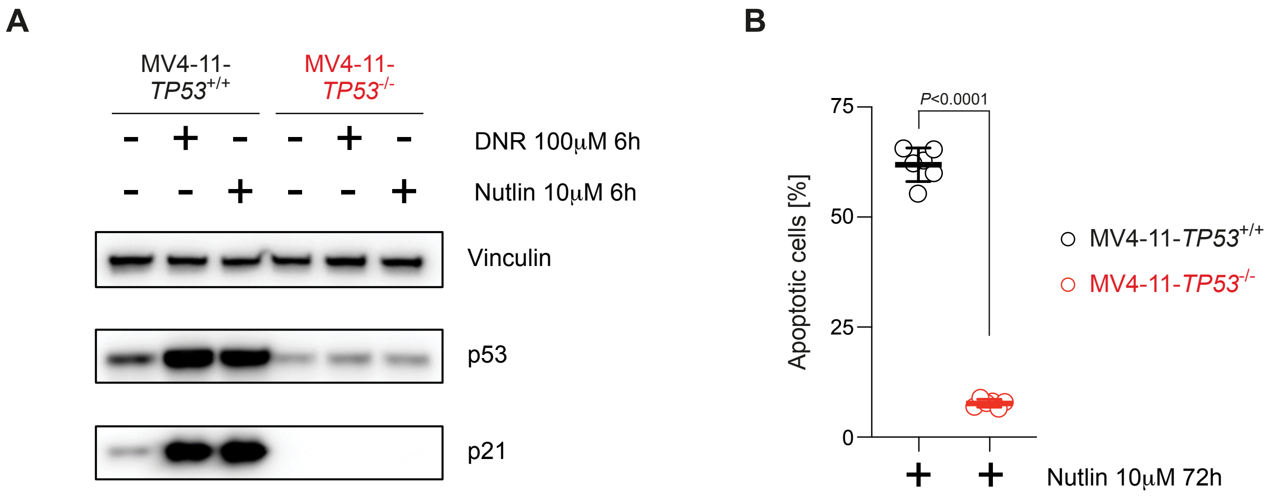

Appendix Table S1: List of antibodies and reagents used for flow cytometry

Target	Clone	Fluorochrome	Supplier	Dilutions
hCD3	OKT3	PE/Dazzle™ 594	BioLegend®	1:600
hCD3	OKT3	APC	BioLegend®	1:400
hCD33	WM53	Brilliant Violet™ 711	BioLegend®	1:600
hCD33	WM53	APC	BD Pharmingen™	1:200
hCD123	6H6	APC	BioLegend®	1:400
hCD279(PD-1)	EH12.2H7	Brilliant Violet421™	BioLegend®	1:300
hCD279(PD-1)	eBioJ205	APC-eFluor™	eBioscience™	1:200
hCD366(TIM3)	F38-2E2	PE	eBioscience™	1:300
hCD366(TIM3)	F38-2E2	PerCP/Cyanine5.5	BioLegend®	1:200
hCD223(LAG3)	11C3C65	Brilliant Violet™ 605	BioLegend®	1:400
hCD274(PD-L1)	29E.2A3	PE/Cyanine7	BioLegend®	1:800
hLDLR	C7	PE	BD Pharmingen™	1:400
hLDLR	301	APC	ThermoFischer	1:200
L/D	NA	Hoechst 33342	Thermo Scientific	1:5’000
L/D	NA	FVD eFluor™ 780	eBioscience™	1:2’000
L/D	NA	DAPI	BioLegend®	1:2’500

Appendix Table S2: Oligos/Primers for introducing Regnase-1 deficiency in CAR T-cells

Name/Target	Sequence (5’ → 3’)	Locus	Direction	Purpose
Z3H12A	AAG GAG GTC TTC TCC TGC CG	Exon 3	NA	crRNA
Z3H12A	GGA ACT GGC ACT GGG AAT GGA	Exon 3	Forward	PCR
Z3H12A	AAT GAC CAC CAT TCA GAG CAG G	Exon 3	Reverse	PCR

Appendix Table S3: Effector-to-target ratios for FACS-sorting and RNA isolation

Effectors	Targets	E :T
CD33 CAR T-cell	MOLM13-*TP53*	1 :8
CD33 CAR T-cell	MV4-11-*TP53*	1 :4
CD123 CAR T-cell	MOLM13-*TP53*	1 :4
CD123 CAR T-cell	MV4-11-*TP53*	1 :1

Appendix figure legends

Appendix Figure S1: Flow cytometry analysis of activation marker surface expression on CD3$^+$ T-cells.

(A) CD25 signal on CD3+ cells for the indicated E:T ratios and days co-incubated with MOLM13-*TP53*$^{+/+}$ AML cells (black) or MOLM13-*TP53*$^{-/-}$ AML cells (red). Y-axes are scaled for every individual measurement.

(B) CD25 signal summary data of individual biological replicates.

Appendix Figure S2: Flow cytometry analysis of exhaustion marker surface expression on CD3$^+$ T-cells.

(A) PD-1, **(B)** LAG3 and **(C)** TIM3 signal on CD3+ cells for the indicated E:T ratios and days co-incubated with MOLM13-*TP53*$^{+/+}$ AML cells (black) or MOLM13-*TP53*$^{-/-}$ AML cells (red). Y-axes are scaled for every individual measurement.

(D) Summary data of exhaustion marker expression of individual biological replicates.

Appendix Figure S3: Flow cytometry analysis of the MOLM13 surface marker CD33 surface signal on CD3$^+$ T-cells.

(A) CD33 signal on CD3+ cells for the indicated E:T ratios and days co-incubated with MOLM13-*TP53*$^{+/+}$ AML cells (black) or MOLM13-*TP53*$^{-/-}$ AML cells (red). Y-axes are scaled for every individual measurement.

(B) CD33 signal summary data of individual biological replicates.

Appendix Figure S4: Flow cytometry analysis of CD33 and PD-L1 on CD3- leukemia cells.

(A) CD33 and **(B)** PD-L1 signal on CD3- leukemia cells for the indicated E:T ratios and days co-incubated with MOLM13-*TP53*$^{+/+}$ AML cells (black) or MOLM13-*TP53*$^{-/-}$ AML cells (red). Y-axes are set at the same maximum for all CD33 and PD-L1 samples, respectively.

Appendix Figure S5: Further details of mRNA-seq data and SREBP gene reporter assays.

(A) Enriched pathways and GO terms relating to BPs for the indicated cell populations in resting MOLM13-*TP53* and **(B)** in T-cells resting or co-incubated with MOLM13-*TP53*.

(C) Luminescent signal of MOLM13-*TP53* and MV4-11-*TP53* AML cells transduced with SREBP-responsive luciferase gene reporter upon incubation with either simvastatin 1μM (for

MOLM13-*TP53*), simvastatin 15μM (for MV4-11-*TP53*) and/or mevalonate 1mM for 1, 4 or 6 days(biological replicates, n=3; 2 technical replicates per biological replicate; symbols indicate individual replicates; thickened lines indicate means and error bars indicate SD; two-way ANOVA).

Appendix Figure S6: Validation of functional p53-deficiency in MV4-11-*TP53* AML cell lines.

(A) Immunoblot for vinculin, p53 and p21 of MV4-11-*TP53* AML cell lines with *TP53*$^{+/+}$ or *TP53*$^{-/-}$ treated with DMSO, Daunorubicin (DNR) 100μM or Nutlin 10μM for 6 hours (3 independent experiments; 1 representative image is shown).

(B) Total percent apoptotic cells of MV4-11-*TP53* AML cell lines treated with DMSO or Nutlin for 72 hours (biological replicates, n=3; 2 technical replicates per biological replicate; symbols indicate individual replicates; thickened lines indicate means and error bars indicate SD; student's t-test).
