# Supplementary material for: Targeting the mevalonate or Wnt pathways to overcome CAR T-cell resistance in TP53-mutant AML cells
Source: EMBO Mol Med. 2024 Feb 14;16(3):3. doi: 10.1038/s44321-024-00024-2 (PMC10940689; doi:10.1038/s44321-024-00024-2)
Supplement: Supplementary file 3 — Movie EV2 [file 44321_2024_24_MOESM3_ESM.zip › Legend Movie EV2_V2.docx]

**Movie EV2**

Representative example of live-cell imaging of an anti-CD33 CAR T-cell (red) engaging a MOLM13-*TP53*^-/-^ AML cell, ultimately leading to AML cell death as visualized by propidium iodide (PI, yellow) influx. Scale bar, 10µm.
