# Supplementary material for: Targeting the mevalonate or Wnt pathways to overcome CAR T-cell resistance in TP53-mutant AML cells
Source: EMBO Mol Med. 2024 Feb 14;16(3):3. doi: 10.1038/s44321-024-00024-2 (PMC10940689; doi:10.1038/s44321-024-00024-2)
Supplement: Supplementary file 10 — Expanded View Figures [file 44321_2024_24_MOESM10_ESM.pdf]

Expanded View Figures

Figure EV1. Details of TP53-associated resistance to in vitro CAR T-cell killing, relates to Figs. 1 and 2.

(**A**) Vector design for second-generation CAR expression under an EF1 promotor with a CD8α hinge, a 4-1BB costimulatory domain, a CD3ζ activating domain and an RQR8 identification and selection peptide. (**B**) Lentiviral T-cell transduction and MACS purification yielded a > 95% pure CAR T-cell population for further experiments. (**C**) In vitro growth kinetics of unperturbed MOLM13-*TP53*$^{+/+}$ and MOLM13-*TP53*$^{-/-}$ leukemia cells. (**D**) Graphical representation of in vitro competitive co-incubation assay. (**E**) Representative FACS plots of in vitro killing assays showing ratio of MOLM13-*TP53*$^{-/-}$GFP^{+} over MOLM13-*TP53*$^{+/+}$RFP^{+} upon 10 days of co-incubation with untransduced T-cell controls and or CAR T-cells. Percentages of parental populations are shown. (**F**) −Log of MOLM13-*TP53*$^{+/+}$/MOLM13-*TP53*$^{-/-}$ ratios normalized to the calculated initial MOLM13-*TP53*$^{+/+}$/MOLM13-*TP53*$^{-/-}$ ratios plotted against time of co-incubation. Pooled results of all different E:T and KO:WT ratios are shown (biological replicates, $n = 2$; three technical replicates per biological replicate; symbols represent means and error bars indicate SD; two-way ANOVA). (**G**) Calculated specific killing from in vitro competitive co-incubation assays of anti-CD33 CAR T-cells against a mixture of MOLM13-*TP53*$^{+/+}$ and MOLM13-*TP53*$^{-/-}$ leukemia cells. (**H**) Absolute CD3^{+} cell numbers at an E:T ratio of 1:16 for untransduced T-cell controls and anti-CD33 CAR T-cells co-incubated with MOLM13-*TP53*$^{+/+}$ (black), MOLM13-*TP53*$^{-/-}$ (red) or MOLM13-*TP53*$^{missense/-}$ (blue) AML cells on day 6 (biological replicates, $n = 2$; two technical replicates per biological replicate; symbols represent individual replicates; thickened lines indicate means and error bars indicate SD; two-way ANOVA). (**I**) CD33 target antigen density on MOLM13-*TP53*$^{+/+}$ and *TP53*$^{-/-}$ AML cells in co-incubation at an E:T of 1:16 with untransduced T-cell controls and anti-CD33-directed CAR T-cells on days 1 and 6 (biological replicates, $n = 4$; three technical replicates for each biological replicates; symbols represent individual replicates; thickened lines indicate means and error bars indicate SD; two-way ANOVA). (**J**) PD-L1 surface expression on MOLM13-*TP53*$^{+/+}$ and *TP53*$^{-/-}$ AML cells in co-incubation with untransduced T-cell controls and anti-CD33-directed CAR T-cells on days 1 and 6 (biological replicates, $n = 3$; two technical replicates for each biological replicate; symbols represent individual replicates; thickened lines indicate means and error bars indicate SD; two-way ANOVA).

© The Author(s)

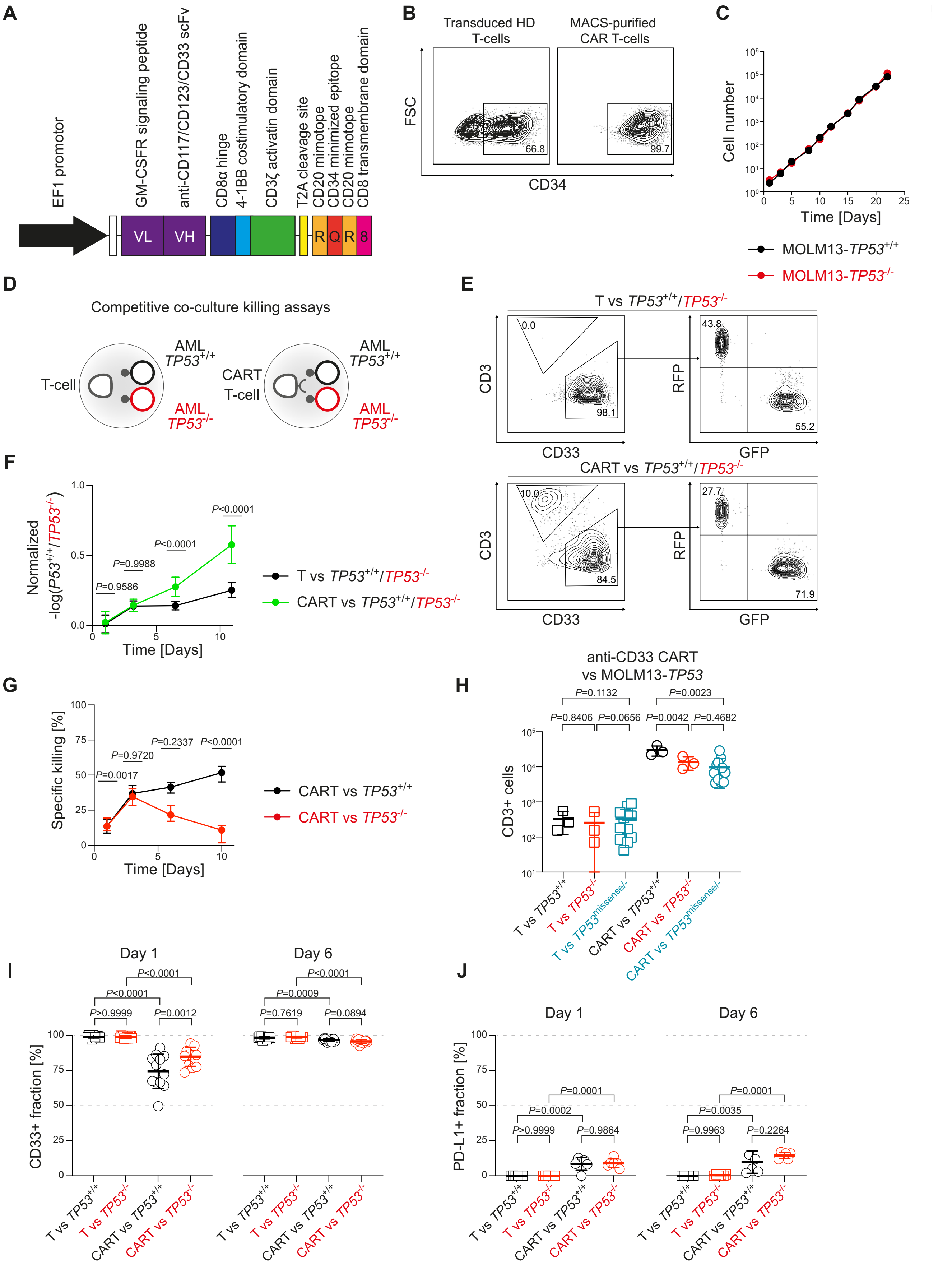

A
EF1 promotor
GM-CSFR signaling peptide
anti-CD117/CD123/CD33 scFv
CD8α hinge
4-1BB costimulatory domain
CD3ζ activatin domain
T2A cleavage site
CD20 mimotope
CD34 minimized epitope
CD20 mimotope
CD8 transmembrane domain
VL VH R Q R 8
B
Transduced HD T-cells
MACS-purified CAR T-cells
FSC
CD34
66.8
99.7
C
Cell number
Time [Days]
MOLM13-TP53+/+
MOLM13-TP53-/-
D
Competitive co-culture killing assays
T-cell
AML TP53+/+
AML TP53-/-
CART T-cell
E
T vs TP53+/+/TP53-/-
CART vs TP53+/+/TP53-/-
CD3
CD33
RFP
GFP
0.0
98.1
43.8
55.2
10.0
84.5
27.7
71.9
F
Normalized -log(P53+/+/TP53-/-)
Time [Days]
P=0.9586
P=0.9988
P<0.0001
P<0.0001
T vs TP53+/+/TP53-/-
CART vs TP53+/+/TP53-/-
G
Specific killing [%]
Time [Days]
P=0.0017
P=0.9720
P=0.2337
P<0.0001
CART vs TP53+/+
CART vs TP53-/-
H
anti-CD33 CART vs MOLM13-TP53
CD3+ cells
P=0.1132
P=0.0023
P=0.8406
P=0.0656
P=0.0042
P=0.4682
T vs TP53+/+
T vs TP53-/-
T vs TP53missense/-
CART vs TP53+/+
CART vs TP53-/-
CART vs TP53missense/-
I
Day 1
Day 6
CD33+ fraction [%]
P<0.0001
P<0.0001
P>0.9999
P=0.0012
P<0.0001
P=0.0009
P=0.7619
P=0.0894
T vs TP53+/+
T vs TP53-/-
CART vs TP53+/+
CART vs TP53-/-
J
Day 1
Day 6
PD-L1+ fraction [%]
P=0.0001
P=0.0002
P>0.9999
P=0.9864
P=0.0001
P=0.0035
P=0.9963
P=0.2264

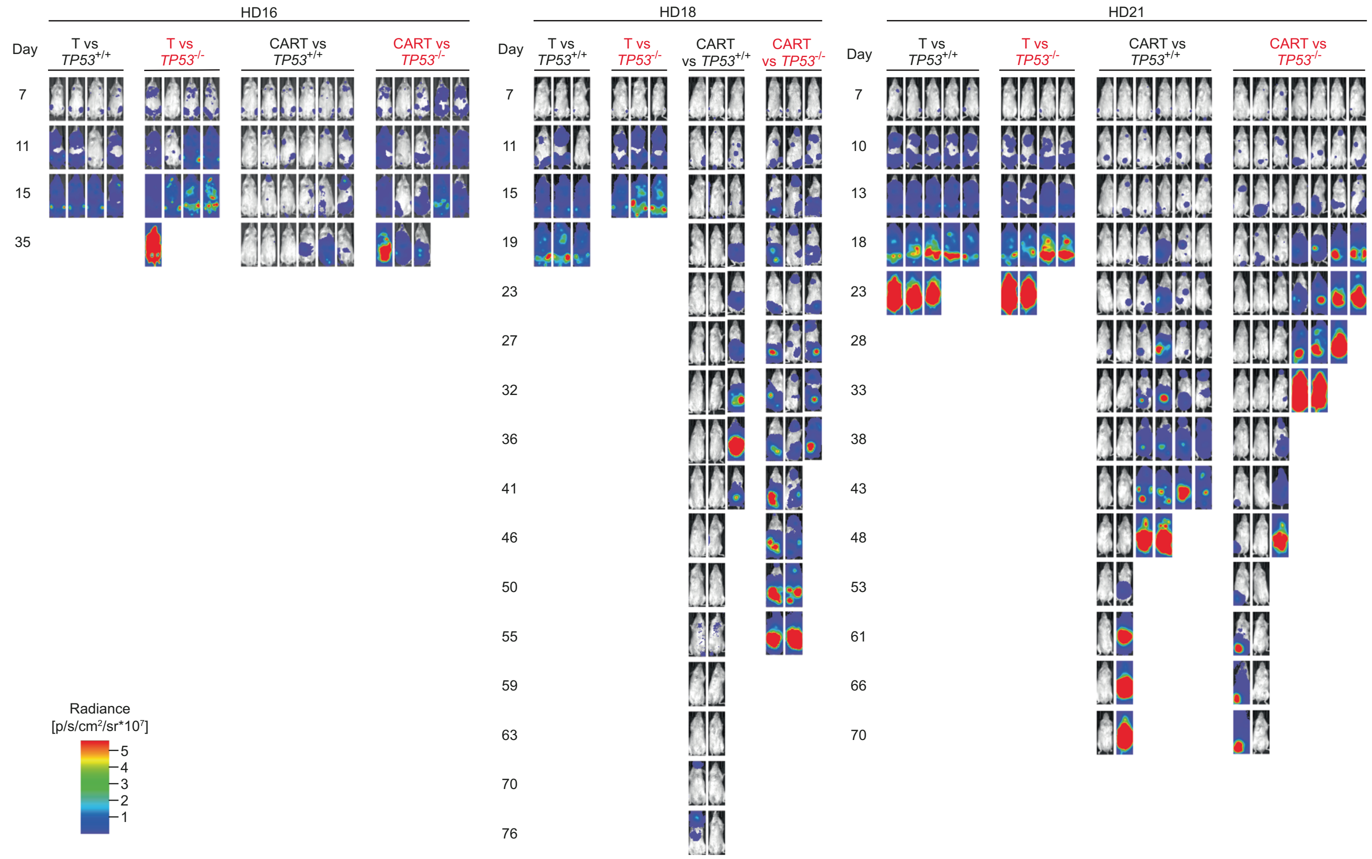

Figure EV2. Details of therapeutic in vivo xenograft model, relates to Fig. 3.

Pseudo-colored bioluminescence measurements taken at the indicated days showing leukemic burden in the respective groups of treated and control mice. The three biological replicates with different HD untransduced T-cells and CAR T-cells are shown, ($n = 53$ mice in total and $n = 3$ biological replicates; T-cells vs. MOLM13-*TP53*$^{+/+}$Luc^{+}=12, T-cells vs. MOLM13-*TP53*$^{-/-}$Luc^{+}=11, CAR T-cells vs. MOLM13-*TP53*$^{+/+}$Luc^{+}=15, CAR T-cells vs. MOLM13-*TP53*$^{-/-}$Luc^{+}=15).

 © The Author(s)

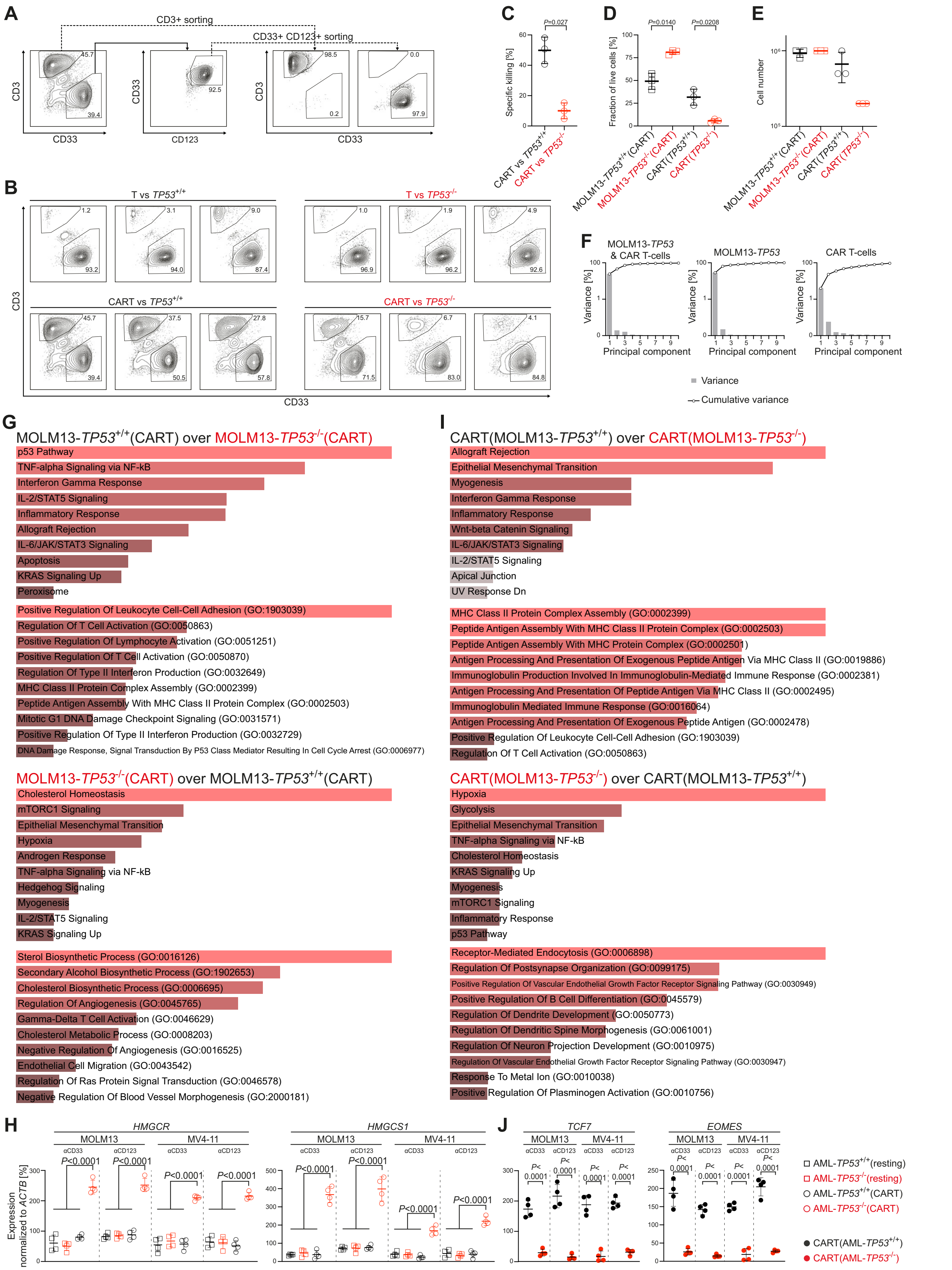

A
CD3+ sorting
CD33+ CD123+ sorting
CD3
CD33
CD123
B
T vs TP53+/+
T vs TP53-/-
CART vs TP53+/+
CART vs TP53-/-
C
Specific killing [%]
P=0.027
CART vs TP53+/+
CART vs TP53-/-
D
Fraction of live cells [%]
P=0.0140
P=0.0208
MOLM13-TP53+/+(CART)
MOLM13-TP53-/-(CART)
CART(TP53+/+)
CART(TP53-/-)
E
Cell number
F
MOLM13-TP53 & CAR T-cells
MOLM13-TP53
CAR T-cells
Variance [%]
Principal component
Variance
Cumulative variance
G
MOLM13-TP53+/+(CART) over MOLM13-TP53-/-(CART)
p53 Pathway
TNF-alpha Signaling via NF-kB
Interferon Gamma Response
IL-2/STAT5 Signaling
Inflammatory Response
Allograft Rejection
IL-6/JAK/STAT3 Signaling
Apoptosis
KRAS Signaling Up
Peroxisome
Positive Regulation Of Leukocyte Cell-Cell Adhesion (GO:1903039)
Regulation Of T Cell Activation (GO:0050863)
Positive Regulation Of Lymphocyte Activation (GO:0051251)
Positive Regulation Of T Cell Activation (GO:0050870)
Regulation Of Type II Interferon Production (GO:0032649)
MHC Class II Protein Complex Assembly (GO:0002399)
Peptide Antigen Assembly With MHC Class II Protein Complex (GO:0002503)
Mitotic G1 DNA Damage Checkpoint Signaling (GO:0031571)
Positive Regulation Of Type II Interferon Production (GO:0032729)
DNA Damage Response, Signal Transduction By P53 Class Mediator Resulting In Cell Cycle Arrest (GO:0006977)
MOLM13-TP53-/-(CART) over MOLM13-TP53+/+(CART)
Cholesterol Homeostasis
mTORC1 Signaling
Epithelial Mesenchymal Transition
Hypoxia
Androgen Response
TNF-alpha Signaling via NF-kB
Hedgehog Signaling
Myogenesis
IL-2/STAT5 Signaling
KRAS Signaling Up
Sterol Biosynthetic Process (GO:0016126)
Secondary Alcohol Biosynthetic Process (GO:1902653)
Cholesterol Biosynthetic Process (GO:0006695)
Regulation Of Angiogenesis (GO:0045765)
Gamma-Delta T Cell Activation (GO:0046629)
Cholesterol Metabolic Process (GO:0008203)
Negative Regulation Of Angiogenesis (GO:0016525)
Endothelial Cell Migration (GO:0043542)
Regulation Of Ras Protein Signal Transduction (GO:0046578)
Negative Regulation Of Blood Vessel Morphogenesis (GO:2000181)
I
CART(MOLM13-TP53+/+) over CART(MOLM13-TP53-/-)
Allograft Rejection
Epithelial Mesenchymal Transition
Myogenesis
Interferon Gamma Response
Inflammatory Response
Wnt-beta Catenin Signaling
IL-6/JAK/STAT3 Signaling
IL-2/STAT5 Signaling
Apical Junction
UV Response Dn
MHC Class II Protein Complex Assembly (GO:0002399)
Peptide Antigen Assembly With MHC Class II Protein Complex (GO:0002503)
Peptide Antigen Assembly With MHC Protein Complex (GO:0002501)
Antigen Processing And Presentation Of Exogenous Peptide Antigen Via MHC Class II (GO:0019886)
Immunoglobulin Production Involved In Immunoglobulin-Mediated Immune Response (GO:0002381)
Antigen Processing And Presentation Of Peptide Antigen Via MHC Class II (GO:0002495)
Immunoglobulin Mediated Immune Response (GO:0016064)
Antigen Processing And Presentation Of Exogenous Peptide Antigen (GO:0002478)
Positive Regulation Of Leukocyte Cell-Cell Adhesion (GO:1903039)
Regulation Of T Cell Activation (GO:0050863)
CART(MOLM13-TP53-/-) over CART(MOLM13-TP53+/+)
Hypoxia
Glycolysis
Epithelial Mesenchymal Transition
TNF-alpha Signaling via NF-kB
Cholesterol Homeostasis
KRAS Signaling Up
Myogenesis
mTORC1 Signaling
Inflammatory Response
p53 Pathway
Receptor-Mediated Endocytosis (GO:0006898)
Regulation Of Postsynapse Organization (GO:0099175)
Positive Regulation Of Vascular Endothelial Growth Factor Receptor Signaling Pathway (GO:0030949)
Positive Regulation Of B Cell Differentiation (GO:0045579)
Regulation Of Dendrite Development (GO:0050773)
Regulation Of Dendritic Spine Morphogenesis (GO:0061001)
Regulation Of Neuron Projection Development (GO:0010975)
Regulation Of Vascular Endothelial Growth Factor Receptor Signaling Pathway (GO:0030947)
Response To Metal Ion (GO:0010038)
Positive Regulation Of Plasminogen Activation (GO:0010756)
H
HMGCR
HMGCS1
MOLM13
MV4-11
αCD33
αCD123
P<0.0001
Expression normalized to ACTB [%]
J
TCF7
EOMES
P< 0.0001
AML-TP53+/+(resting)
AML-TP53-/-(resting)
AML-TP53+/+(CART)
AML-TP53-/-(CART)
CART(AML-TP53+/+)
CART(AML-TP53-/-)

Figure EV3. Details of sorting procedure and gene expression profiling results, relates to Fig. 4.

(**A**) Gating strategy for flow cytometry sorting as shown by an example (i.e., same data) from panel (**B**): $CD3^+$ T-cells were sorted as shown, $CD3^-CD33^+$ events were further gated on $CD33^+CD123^+$ to sort double-positive MOLM13-*TP53* AML leukemia cells. The resulting cell populations of >97% purity are shown on the right two panels. (**B**) FACS plots of $n = 3$ technical replicates of in vitro co-culture assays subjected to flow cytometry sorting and mRNA extraction. (**C**) Calculated specific killing from co-incubation assay of MOLM13-$TP53^{+/+}$ (black) or MOLM13-$TP53^{-/-}$ (red) with anti-CD33 CAR T-cells used for RNA-sequencing (biological replicate, $n = 1$; three technical replicates; symbols indicate individual technical replicates; thickened line represents mean and error bars indicate SD; unpaired Student's *t* test). (**D**) Fraction of live cells from co-incubation assay used for RNA-sequencing. (biological repliactes, $n = 1$; 3 technical replicates; symbols represent individual replicates; thickened lines indicate mean and error bars represent SD; unpaired Student's *t* test). (**E**) Total sorted cell numbers for the respective conditions used for mRNA sequencing. (**F**) Proportion of variance plot. Bars represent the specific proportion of total variance explained by the principal component (PC) and curve represents the cumulative variance explained by PC and all PCs before it. (**G**) Pathways and gene ontology (GO) terms related to biological processes (BPs) enriched in MOLM13-*TP53* under CAR T-cell attack. (**H**) RT-qPCR measuring expression of *HMGCR* and *HMGCS1* transcripts normalized to *ACTB* in MOLM13-*TP53* and MV4-11-*TP53* cells sorted from co-incubation assays with either untransduced T-cells [AML-*TP53*(resting)] or CD33-/CD123-directed CAR T-cells [AML-*TP53*(CART)] (biological replicates, $n = 2$; 2 technical replicates per biological replicate; symbols indicate individual replicates; thickened lines indicate mean and error bars indicate SD; one-way ANOVA). (**I**) Pathways and gene ontology (GO) terms related to biological processes (BPs) enriched in CAR T-cells co-incubated with MOLM13-*TP53*. **(J)** RT-qPCR measuring expression of *TCF7* and *EOMES* transcripts normalized to *ACTB* in CAR T-cells sorted from co-incubation assays with $TP53^{+/+}$ [CART(AML-$TP53^{+/+}$]) or $TP53^{-/-}$ [CART(AML-$TP53^{-/-}$]) target cells lines MOLM13 and MV4-11 (biological replicates, $n = 2$; 2 technical replicates per biological replicate; symbols indicate individual replicates; thickened lines indicate mean and error bars indicate SD; unpaired Student's *t* test).

 © The Author(s)

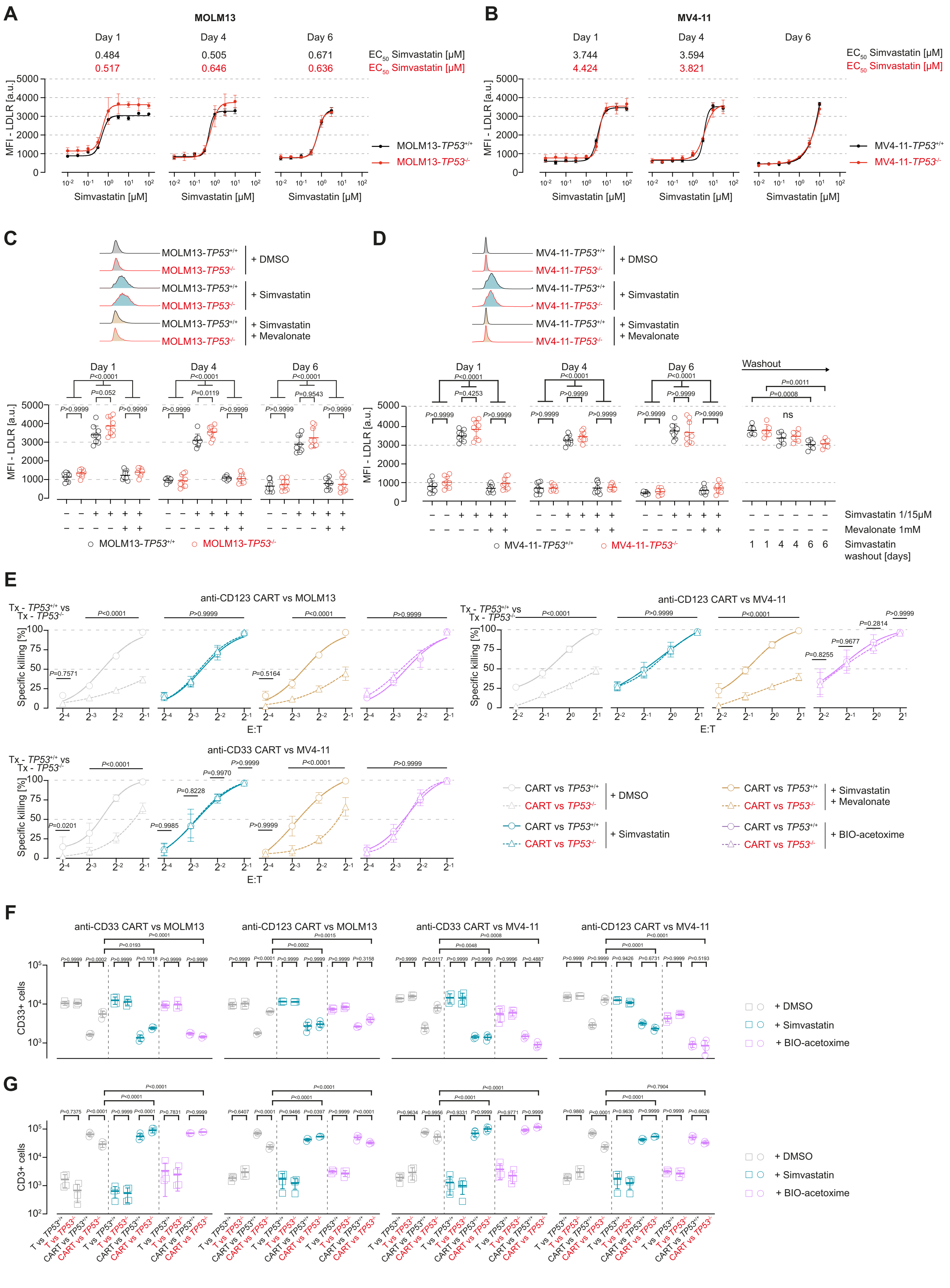
A
MOLM13
Day 1
Day 4
Day 6
0.484
0.517
0.505
0.646
0.671
0.636
EC50 Simvastatin [µM]
EC50 Simvastatin [µM]
MFI - LDLR [a.u.]
Simvastatin [µM]
MOLM13-TP53+/+
MOLM13-TP53-/-
B
MV4-11
3.744
4.424
3.594
3.821
MV4-11-TP53+/+
MV4-11-TP53-/-
C
MOLM13-TP53+/+
MOLM13-TP53-/-
+ DMSO
+ Simvastatin
+ Simvastatin
+ Mevalonate
D
MV4-11-TP53+/+
MV4-11-TP53-/-
Washout
ns
Simvastatin 1/15µM
Mevalonate 1mM
Simvastatin washout [days]
E
anti-CD123 CART vs MOLM13
anti-CD123 CART vs MV4-11
anti-CD33 CART vs MV4-11
Tx - TP53+/+ vs
Tx - TP53-/-
Specific killing [%]
E:T
CART vs TP53+/+
CART vs TP53-/-
+ DMSO
+ Simvastatin
+ Simvastatin
+ Mevalonate
+ BIO-acetoxime
F
anti-CD33 CART vs MOLM13
anti-CD123 CART vs MOLM13
anti-CD33 CART vs MV4-11
anti-CD123 CART vs MV4-11
CD33+ cells
+ DMSO
+ Simvastatin
+ BIO-acetoxime
G
CD3+ cells
T vs TP53+/+
T vs TP53-/-
CART vs TP53+/+
CART vs TP53-/-

© The Author(s)

Figure EV4. Details of rescue co-incubation assays, relates to Figs. 5 and 6.

LDLR mean fluorescence intensity and extrapolated EC50 of (**A**) MOLM13-*TP53* and (**B**) MV4-11-*TP53* AML cells incubated with increasing concentrations of simvastatin (biological replicates, $n = 2$; symbols represent means; error bars indicate SD). (**C**) Upper panel: Representative FACS histograms of LDLR mean fluorescence intensity of isogenic MOLM13-*TP53* AML cells with wild-type (MOLM13-$TP53^{+/+}$) or null (MOLM13-$TP53^{-/-}$) *TP53* status in the presence of DMSO, simvastatin 1 µM or simvastatin 1 µM + mevalonate 1mM. Lower panel: LDLR mean fluorescence intensity of MOLM13-*TP53* cells on days 1, 4 and 6 in the presence or absence of simvastatin 1 µM and/or mevalonate 1mM (biological replicates, $n = 3$; 2 technical replicates per biological replicate; symbols indicate individual replicates; thickened lines indicate means and error bars indicate SD; two-way ANOVA). (**D**) Upper panel: Representative FACS histograms of LDLR mean fluorescence intensity of isogenic MV4-11-*TP53* AML cells with wild-type (MV4-11-$TP53^{+/+}$) or null (MV4-11-$TP53^{-/-}$) *TP53* status in the presence of DMSO, simvastatin 15 µM or simvastatin 15 µM + mevalonate 1mM. Lower panel: LDLR mean fluorescence intensity of MV4-11-*TP53* cells on days 1, 4 and 6 in the presence or absence of simvastatin 15 µM and/or mevalonate 1mM as well as after washout of simvastatin (biological replicates, $n = 3$; 2 technical replicates per biological replicate; symbols indicate individual replicates; thickened lines indicate means and error bars indicate SD; two-way ANOVA). (**E**) Summary data showing results from 3 different co-incubation assays of CAR T-cell-mediated killing of $TP53^{+/+}$ or $TP53^{-/-}$ AML cells over various E:T ratios in the presence of DMSO, simvastatin (1 µM for MOLM13 and 15 µM for MV4-11), simvastatin + mevalonate 1mM or BIO-acetoxime 0.5 µM, respectively. Co-incubation assays include anti-CD123 CAR vs MOLM13-*TP53*, anti-CD33 CAR vs MV4-11-*TP53* and anti-CD123 CAR vs MV4-11-*TP53*. (replicates, $n = 2$–3; symbols represent mean; error bars indicate SD; two-way ANOVA). (**F**) Absolute CD33+ target cell numbers from various co-incubation assays of untransduced T-cells or CAR T-cells with $TP53^{+/+}$ or $TP53^{-/-}$ AML cells at an E:T of 1:16 in the presence of DMSO, simvastatin (1 µM for MOLM13 and 15 µM for MV4-11) or BIO-acetoxime 0.5 µM, respectively. Co-incubation assays include anti-CD33 CAR vs MOLM13-*TP53*, anti-CD123 CAR vs MOLM13-*TP53*, anti-CD33 CAR vs MV4-11-*TP53* and anti-CD123 CAR vs MV4-11-*TP53*. (biological replicates, $n = 2$; 2 technical replicates per biological replicate; symbols represent individual replicates; thickened lines indicate means and error bars indicate SD; ns, non-significant; two-way ANOVA). (**G**) Absolute CD3+ effector cell numbers from various co-incubation assays of untransduced T-cells or CAR T-cells with $TP53^{+/+}$ or $TP53^{-/-}$ AML cells at an E:T of 1:16 in the presence of DMSO, simvastatin (1 µM for MOLM13 and 15 µM for MV4-11) or BIO-acetoxime 0.5 µM, respectively. Co-incubation assays include anti-CD33 CAR vs MOLM13-*TP53*, anti-CD123 CAR vs MOLM13-*TP53*, anti-CD33 CAR vs MV4-11-*TP53* and anti-CD123 CAR vs MV4-11-*TP53*. (biological replicates, $n = 2$; 2 technical replicates per biological replicate; symbols represent individual replicates; thickened lines indicate means and error bars indicate SD; two-way ANOVA).

 © The Author(s)

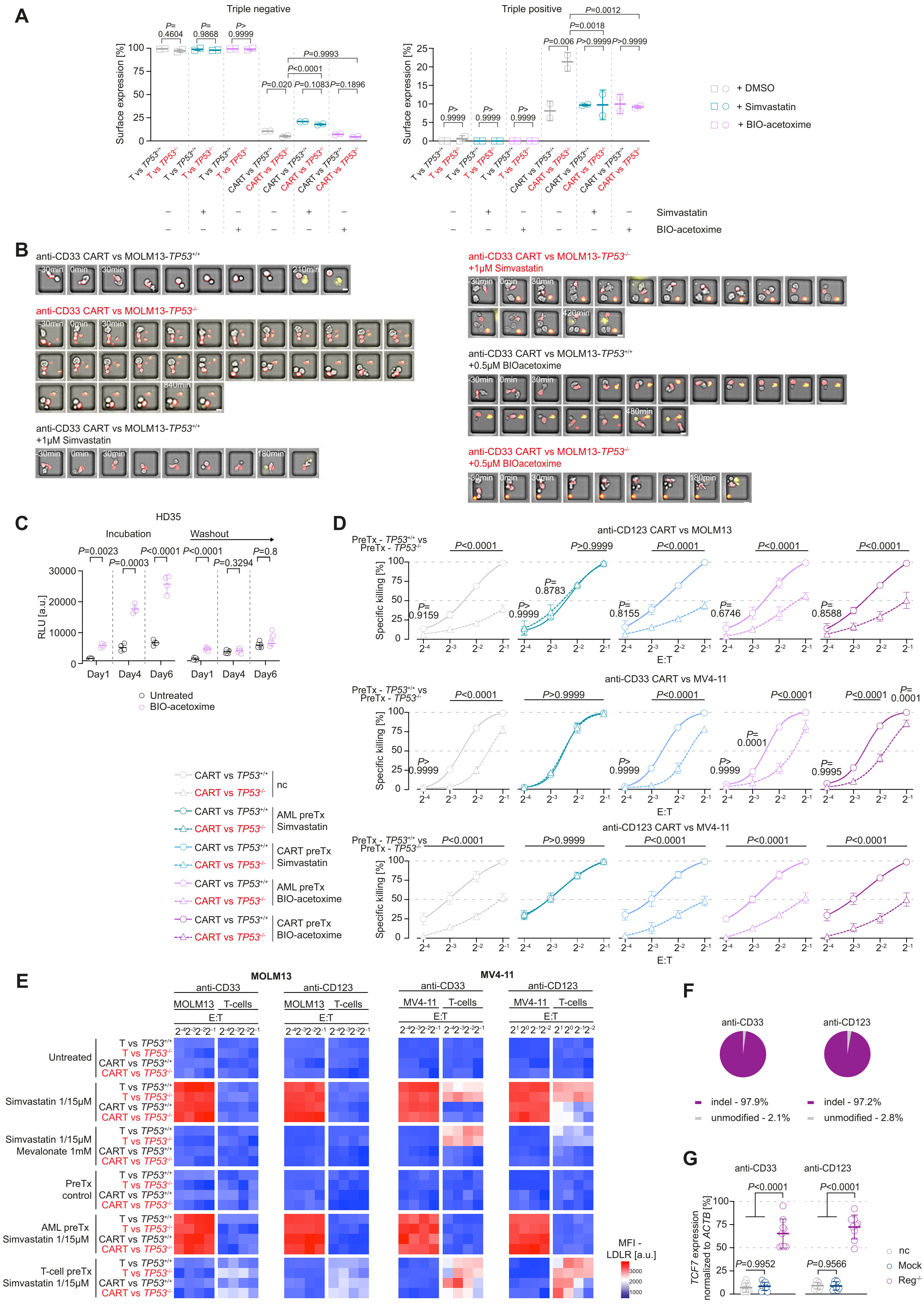

A
Triple negative
Triple positive
Surface expression [%]
+ DMSO
+ Simvastatin
+ BIO-acetoxime
Simvastatin
BIO-acetoxime
B
anti-CD33 CART vs MOLM13-TP53+/+
anti-CD33 CART vs MOLM13-TP53-/-
anti-CD33 CART vs MOLM13-TP53+/+ +1µM Simvastatin
anti-CD33 CART vs MOLM13-TP53-/- +1µM Simvastatin
anti-CD33 CART vs MOLM13-TP53+/+ +0.5µM BIOacetoxime
anti-CD33 CART vs MOLM13-TP53-/- +0.5µM BIOacetoxime
C
HD35
Incubation
Washout
RLU [a.u.]
Day1
Day4
Day6
Untreated
BIO-acetoxime
D
anti-CD123 CART vs MOLM13
anti-CD33 CART vs MV4-11
anti-CD123 CART vs MV4-11
PreTx - TP53+/+ vs PreTx - TP53-/-
Specific killing [%]
E:T
CART vs TP53+/+
CART vs TP53-/-
nc
AML preTx Simvastatin
CART preTx Simvastatin
AML preTx BIO-acetoxime
CART preTx BIO-acetoxime
E
MOLM13
MV4-11
anti-CD33
anti-CD123
T-cells
Untreated
Simvastatin 1/15µM
Simvastatin 1/15µM Mevalonate 1mM
PreTx control
AML preTx Simvastatin 1/15µM
T-cell preTx Simvastatin 1/15µM
MFI - LDLR [a.u.]
F
anti-CD33
anti-CD123
indel - 97.9%
unmodified - 2.1%
indel - 97.2%
unmodified - 2.8%
G
TCF7 expression normalized to ACTB [%]
nc
Mock
Reg-/-

Figure EV5. Details of rescue and pretreatment co-incubation assays as well as Regnase-1-deficient CAR T-cells, relates to Figs. 5 and 6.

(**A**) Fraction of T-cells negative for all three investigated exhaustion markers (right) and negative for all three markers (left) treated with the indicated compounds (one biological replicate, $n = 2$ technical replicates; symbols represent means; error bars indicate SD; two-way ANOVA). (**B**) Representative stills of fluorescence live-cell time-lapse imaging data of anti-CD33 CAR T-cell engaging MOLM13-*TP53*$^{+/+}$ AML or MOLM13-*TP53*$^{-/-}$ AML cells in the presence of simvastatin 1 μM or BIO-acetoxime 0.5 μM (scale bars, 10μm). (**C**) Luminescence signal of T-cells transduced with a Wnt-responsive luciferase gene reporter system and incubated with or without BIO-acetoxime at 0.5 μM for 1, 4 or 6 days as well as after washout of BIO-acetoxime. (Data shown from a second healthy T-cell donor than in Fig. 6C; biological replicates, $n = 2$; 2 technical replicates per biological replicate; symbols indicate individual replicates; thickened lines denote means and error bars indicate SD; unpaired Student's *t* test). (**D**) Results from 3 different pretreatment co-incubation assays with anti-CD33 and anti-CD123 CAR T-cells against *TP53*$^{+/+}$ or *TP53*$^{-/-}$ AML (MOLM13 and MV4-11) cells at various E:T ratios. Co-incubations include: anti-CD123 CAR vs MOLM13-*TP53*, anti-CD33 CAR vs MV4-11-*TP53* and anti-CD33 CAR vs MV4-11-*TP53*. (biological replicates, $n = 2$–3; 2 technical replicates per biological replicate; symbols represent means; error bars indicate SD; two-way ANOVA). **(E)** Heatmaps depicting LDLR expression (mean fluorescence intensity) within treatment as well as pretreatment co-incubation assays with anti-CD33 and anti-CD123 CAR T-cells against MOLM13-*TP53* and MV4-11-*TP53* AML cells at various E:T ratios. (biological replicates, $n = 3$–4; 2 technical replicates per biological replicate; pseudocolors indicate signal of LDLR expression). (**F**) Sequencing results graphed as %indels of Regnase-1 CRISPRed anti-CD33 as well as anti-CD123 CAR T-cells from one healthy donor. (**G**) RT-qPCR measuring expression of *TCF7* transcripts normalized to *ACTB* in nc, mock and RegKO anti-CD33 and anti-CD123 CAR T-cells (biological replicates, $n = 2$; 2 technical replicates per biological replicate; symbols indicate individual replicates; thickened lines indicate means and error bars indicate SD; paired Student's *t* test).

 © The Author(s)
